# Evidence that pairing with genetically similar mates is maladaptive in a monogamous bird

**DOI:** 10.1186/1471-2148-9-147

**Published:** 2009-06-30

**Authors:** Hervé Mulard, Etienne Danchin, Sandra L Talbot, Andrew M Ramey, Scott A Hatch, Joël F White, Fabrice Helfenstein, Richard H Wagner

**Affiliations:** 1 Laboratoire Fonctionnement et Évolution des Système Écologiques, CNRS-UMR 7103, Ecology Institute, Université Pierre et Marie Curie-Paris 6, 7 Quai St Bernard, 75005 Paris, France; 2 Konrad Lorenz Institute for Ethology, Austrian Academy of Sciences, Savoyenstrasse 1a, A-1160 Vienna, Austria; 3 Laboratoire Évolution et Diversité Biologique, UMR 5174, Université Paul Sabatier, 118 Route de Narbonne, 31962 Toulouse Cedex 9, France; 4 U.S. Geological Survey, Alaska Science Center, 4210 University Drive, Anchorage, Alaska, 99508, USA; 5 Evolutionary Ecology Group, Institute of Ecology and Evolution, University of Bern, Baltzerstrasse 6, 3012 Bern, Switzerland; 6 Laboratoire d'Écologie et de Neuro-Éthologie Sensorielles, Université Jean Monnet, 23 Rue Paul Michelon, 42023 Saint-Étienne Cedex 03, France

## Abstract

**Background:**

Evidence of multiple genetic criteria of mate choice is accumulating in numerous taxa. In many species, females have been shown to pair with genetically dissimilar mates or with extra-pair partners that are more genetically compatible than their social mates, thereby increasing their offsprings' heterozygosity which often correlates with offspring fitness. While most studies have focused on genetically promiscuous species, few studies have addressed genetically monogamous species, in which mate choice tends to be mutual.

**Results:**

Here, we used microsatellite markers to assess individual global heterozygosity and genetic similarity of pairs in a socially and genetically monogamous seabird, the black-legged kittiwake *Rissa tridactyla*. We found that pairs were more genetically dissimilar than expected by chance. We also identified fitness costs of breeding with genetically similar partners: (i) genetic similarity of pairs was negatively correlated with the number of chicks hatched, and (ii) offspring heterozygosity was positively correlated with growth rate and survival.

**Conclusion:**

These findings provide evidence that breeders in a genetically monogamous species may avoid the fitness costs of reproducing with a genetically similar mate. In such species that lack the opportunity to obtain extra-pair fertilizations, mate choice may therefore be under high selective pressure.

## Background

Numerous traits influence mate choice that may produce non-random mating patterns in many species. Although most studies have focused on morphological and behavioral traits [1-4]), there is rapidly growing evidence of multiple genetic criteria of mate choice (reviewed in [5,6]). For example, females might choose the most heterozygous males [7], which may increase the resistance of offspring to parasites [8,9]. Alternatively, females might choose males carrying alleles that are compatible with their own genotypes. The main driving forces of mate choice would then be to maintain equilibrium between co-adapted genes [10] or alternatively, to enhance the genetic variability of offspring [11-16].

In species with biparental care, both sexes should be choosy in selecting a mate [17,18]. Blomqvist et al. [14] reported that in socially monogamous shorebirds, both sexes obtained extra-pair fertilizations when mates were genetically similar. In blue tits (*Parus caeruleus*), females acquired extra-pair fertilizations that enhanced the heterozygosity and fitness of their offspring [19]. In superb starlings (*Lamprotornis superbus*), the benefits of extra-pair fertilizations may differ according to the genetic similarity of the extra-pair partner [20]. However, few studies have focused on the mating patterns in genetically monogamous species which lack extra-pair fertilizations to diminish the costs of pairing with a suboptimal mate.

The black-legged kittiwake (*Rissa tridactyla*) is a long-lived, monogamous seabird with no extra-pair fertilizations [21] and high between-year repairing rates [22-25]. In this species, mate choice may thus profoundly affect reproductive success throughout an individual's lifetime. To examine whether mating patterns in kittiwakes are influenced by genetic criteria, we assessed individual heterozygosity and genetic similarity of mates with microsatellite markers.

Our first aim was to examine three hypotheses of mating patterns driven by individual genotypes. Breeders may be paired with: (1) heterozygous mates ("preference of heterozygous mates" hypothesis; [15]), (2) genetically dissimilar mates, in order to increase the genetic variability of offspring ("genetic similarity avoidance" hypothesis; [14,26,27]), or (3) genetically similar mates, in order to preserve the link between locally co-adapted genes ("genetic similarity preference" hypothesis; [10,28]). According to Hypothesis 1, the most heterozygous mates are of better quality because they may provide direct benefits (e.g., better parental care) and/or indirect benefits in term of more heterozygous offspring. The hypothesis predicts that paired individuals are more heterozygous than unpaired ones, and that there a positive correlation between male and female heterozygosity [7]. Hypothesis 2 predicts that the observed mean genetic similarity between pair members is lower than expected through random matings. Hypothesis 3 is the reverse of Hypothesis 2 and predicts that mates share more alleles than expected by chance, meaning that the observed mean genetic similarity between pair members will be higher than expected through random matings.

Our second aim was to examine the fitness consequences of breeding with genetically similar or dissimilar individuals. We searched for possible relationships between genetic characteristics of pairs and fitness components such as clutch size and hatching success [29,30]. Because genetically similar pairs are more likely to produce homozygous offspring than dissimilar pairs, we also examined predicted relationships between offspring heterozygosity and their growth and survival.

Microsatellites are generally assumed to be neutral genetic markers and have been widely used to estimate relatedness, individual heterozygosity and population level of inbreeding [31-34]. If heterozygosity at certain selected loci enhances fitness [8,15], heterozygosity at microsatellite loci may be a good surrogate of the overall genetic quality of an individual, especially in wild species where little is known about genes under selection. However, Lieutenant-Gosselin & Bernatchez [35] and Tiira et al. [36] have shown that global heterozygosity might be highly influenced by heterozygosity at certain specific loci. Such loci may be physically linked to fitness loci, and one should therefore distinguish effects of global heterozygosity from effects of heterozygosity at each microsatellite marker. Thus we performed our analyses both with the global heterozygosity and with the heterozygosity at each locus in order to distinguish the effects of global heterozygosity from that of specific loci that might be physically linked to fitness loci.

## Results

We monitored 348 genotyped adults in 2003 and 2004: 241 were seen alive in 2003 and 289 in 2004. Adults formed 74 pairs in 2003 and 72 in 2004; the remaining adults for each year corresponded to unpaired adults or adults paired with non-genotyped mates. All these adults were included in the bootstrap analyses because they were alive in the considered year and thus potentially available for pairing.

For chicks with genotyped parents, we found that *Phm*_*xy *_of the parents was closely related to all indices of offspring heterozygosity (82 chicks, p < 0.007), confirming that *Phm*_*xy *_might be a reliable estimate of the probability of a given pair of producing homozygous offspring. Furthermore, *Phm*_*xy *_of pairs used in our study exhibited a wide, six-fold range of variation (from 0.06 to 0.39, mean: 0.18 +/- 0.06).

### Mating pattern and genetics

To correct for linkages between loci, OHW loci and K31 were excluded from calculations of *H*, *SH *and *IR*, but results did not differ with all loci. The "preference of heterozygous mates" hypothesis (hypothesis 1) predicted assortative mating by heterozygosity. However, male and female *H *were not correlated for any year (r ranging from 0.021 to 0.10, p ranging from 0.38 to 0.86). Results were the same for *SH *(0.02 ≤ r ≤ 0.13, 0.26 ≤ p ≤ 0.87) and *IR *(0.05 ≤ r ≤ 0.10, 0.38 ≤ p ≤ 0.67). Heterozygosity of paired and unpaired individuals did not differ (for all indices: in 2003, t_2,241 _< 1.93, p > 0.06; in 2004, t_2,241 _< 1.25, p > 0.21).

**Figure 1:**
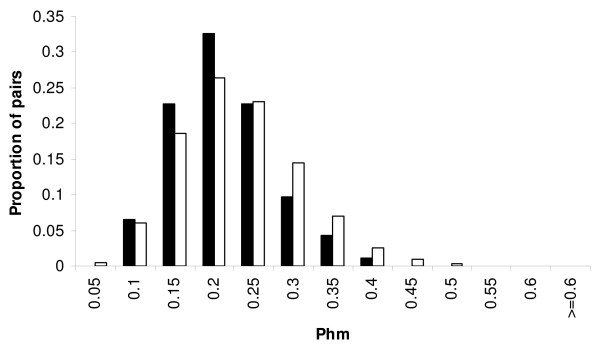
**Distribution of genetic similarity (calculated with the *Phm *index) in pairs observed in 2003 and/or 2004**. Black bars represent the percentage of observed pairs (n = 92) and white bars represent a random distribution of the *Phm *of pairs obtained by simulating 10,000 pairings. *Phm *was calculated with all ten loci. Kittiwake pairs were less genetically similar than expected by chance (p = 0.016).

The "genetic similarity avoidance" hypothesis (hypothesis 2) predicted that observed pairs comprise less genetically similar individuals than expected by chance. To test this, for each year we ran 10,000 bootstraps using the observed individuals. For each run, we used either a calculation across all loci, or excluding OHW loci (Table 1). We found a significant difference in terms of genetic similarity between observed and simulated pairs in 2003 and 2004 (Figure 1), indicating that pairs were formed of more genetically dissimilar individuals than expected by chance.

**Table 1:** Differences between observed and simulated means of genetic similarity of mates

	***Phm ***(prob. of producing a homozygous offspring)			
	
	**All pairs in both years**	**Stable pairs in both years**	**2003**	**2004**
*Pairs (P)*	92	58	74	72
				
*Adults (N)*	348	348	241	289

All loci	0.184	0.182	0.181	0.184
	0.199	0.199	0.196	0.201
	**0.016**	**0.029**	**0.021**	**0.018**

Without OHW loci	0.172	0.171	0.162	0.174
	0.189	0.189	0.184	0.191
	**0.026**	**0.048**	**0.005**	**0.043**

Distributions of *Phm *estimated by performing 10,000 random repairings of *P *pairs among the *N *possible adults and calculating the simulated mean genetic similarity between mates. For each estimate, the first provides the observed mean genetic similarity between mates for the population and the second line reports the simulated mean, the last line is the p-value calculated as the proportion of bootstraps having a mean genetic similarity between mates lower than the observed mean. Significant p-values are in bold. The first column contains the results for both years combined (each pair counted only once), the second contains the results for all years combined using only pairs breeding together in both years (with again each pair counted only once).

When pooling pairs seen in 2003 and 2004 (one observation for each pair, column "all pairs" in Table 1), we still found a significant difference in genetic dissimilarity between observed and simulated pairs. Results were similar when taking only pairs of kittiwakes breeding together both years (considered as "stable pairs", Table 1). Because of a low divorce rate, we lacked a sufficient sample to compare the genetic similarity of divorced versus reunited pairs.

The "genetic similarity preference" hypothesis (hypothesis 3) predicts that mates share more alleles than expected by chance. This means that observed pairs should comprise more genetically similar individuals than expected by chance. We would then expect to have a p-value for *Phm*_*xy *_higher than 0.95, which we did not find (Table 1).

### Reproductive success and genetic similarity

**Figure 2:**
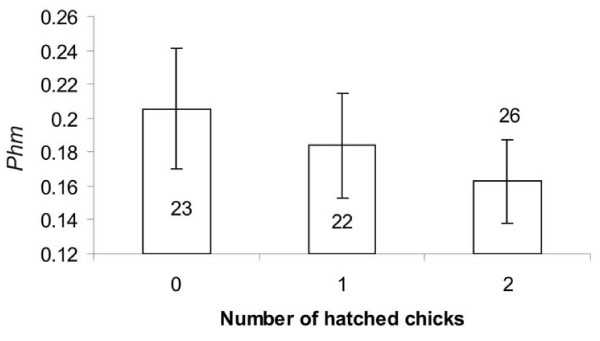
**Relationship between number of hatched chicks and *Phm *in 2003**. Here, the probability of producing a homozygous offspring (*Phm*) was calculated excluding out of Hardy-Weinberg (OHW) loci, but results were similarly significant with all ten loci. The number of hatched chicks decreased significantly with genetic similarity of pairs (see the text for statistical tests).

We used only 2003 data to determine the number of eggs laid and the number and proportion of eggs hatched, because the reproductive success of some of the pairs in 2004 and subsequent years may have been affected by other experiments after pair formation. Genetic similarity indices were computed without OHW loci and K31. The number of eggs laid was not correlated with pair genetic similarity (χ^2^_72 _= 0.01, p = 0.91, n = 74). However, for pairs that laid eggs, the number of hatched chicks was correlated with *Phm*_*xy *_(χ^2^_69 _= 4.0, p = 0.045, n = 71), with the mean number of chicks hatched being lower in more genetically similar pairs (Figure 2). Similarly, hatching rate was also negatively correlated with *Phm*_*xy *_(χ^2^_69 _= 4.0, p = 0.045, n = 71).

### Offspring growth and survival in relation to offspring heterozygosity

**Figure 3:**
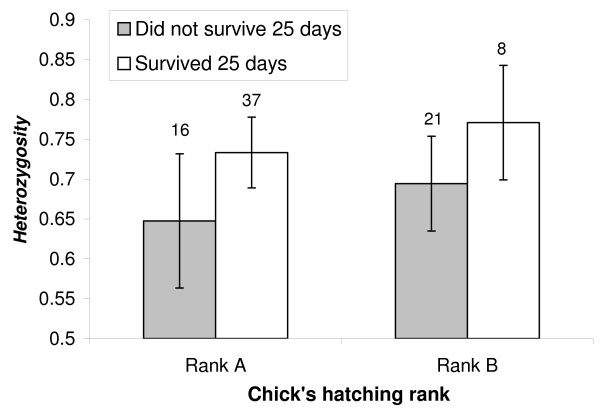
**Relationship between heterozygosity and chick survival in 2005**. We show the correlation between chick survival up to 25 days of age and chick heterozygosity estimated by the *H *index calculated over all loci. Chicks that survived 25 days were significantly more heterozygous than chicks that died (see the text for statistical tests).

Chicks were not genotyped for K67 (because of low variability). In 2005, we assessed the survival and growth in body weight, tarsus and wing length of 82 chicks until age 25 days. These life history parameters were highly correlated to hatching rank (χ^2 ^> 12, p < 0.001 in all analyses). The hatching rank*chick heterozygosity interactions were non-significant for all heterozygosity indices (p > 0.6) and were thus discarded from the models. Chick survival was positively correlated with chick heterozygosity for all indices (H: χ^2 ^= 5.2, p = 0.022, see Figure 3; SH: χ^2 ^= 4.7, p < 0.03; IR: χ^2 ^= 4.5, p = 0.03), but became non-significant when removing OHW loci and K31 from the computation of chick heterozygosity (H: χ^2 ^= 3.6, p = 0.06; SH: χ^2 ^= 2.5, p = 0.11; IR: χ^2 ^= 3.3, p = 0.07).

**Figure 4:**
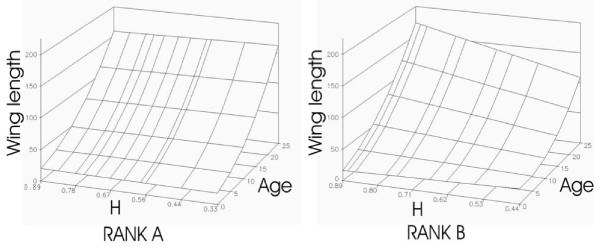
**Relationship between chick heterozygosity, age and growth rate in 2005**. We plotted the predicted value of chick growth in wing length (in mm) according to the model A+H+R+AH+AR+HR+ARH+A^2 ^where A is the age of the chick (measured in days), R its hatching rank (binary factor equaling A for first hatched, or B for second hatched chicks) and H its heterozygosity (according to the *H *index). For clarity, the random parameter (chick's identity) was removed from the model. Significance of the statistical tests is given in Table 2.

When analyzing chick growth in body weight, wing and tarsus length, we found a significant interaction of Age*Chick heterozygosity*Hatching rank (p < 0.017, when chick heterozygosity was calculated over all loci, p < 0.089 when chick heterozygosity was calculated without OHW loci and K31; Table 2 and Additional file 1). This effect of chick heterozygosity on chick growth was however only evident for B-chicks (Table 2, see Figure 4 for a representation of this effect on chick growth in wing length).

**Table 2:** Relationships between chick age, hatching rank, heterozygosity and growth in body weight, wing and tarsus length

Explained variable	Hz index used	A*H*R	AIC	A*H in A-chicks	A*H in B-chicks
*Body weight*	*H*	**<0.0001**	3267.5	0.68	**<0.0001**
	
	*SH*	**<0.0001**	3271.7	0.44	**<0.0001**
	
	*IR*	**<0.0001**	3273.3	0.86	**<0.0001**
	
	*H'*	**0.020**	3284.8	0.12	**0.0020**
	
	*SH'*	**0.046**	3285.9	0.094	**0.0038**
	
	*IR'*	0.064	3289.2	0.12	**0.0055**

*Wing length*	*H*	**<0.0001**	2612.3	0.58	**<0.0001**
	
	*SH*	**0.0001**	2615.0	0.91	**<0.0001**
	
	*IR*	**<0.0001**	2615.4	0.39	**<0.0001**
	
	*H'*	**0.013**	2621.7	0.31	**0.0007**
	
	*SH'*	**0.037**	2621.6	0.15	**0.0013**
	
	*IR'*	**0.038**	2626.8	0.41	**0.0033**

*Tarsus length*	*H*	**0.0017**	2758.8	0.71	**0.0025**
	
	*IR*	**0.0013**	2760.4	0.95	**0.011**
	
	*SH*	**0.012**	2762.2	0.28	**0.0045**
	
	*H'*	**0.027**	2765.2	0.75	**0.04**
	
	*IR'*	**0.014**	2767.2	0.20	0.054
	
	*SH'*	0.089	2766.3	0.97	0.089

We used AIC for model selection. Parameters: A (Age), H (Heterozygosity, estimated by the index in the column "Hz index used") and R (hatching Rank, a binary effect). For heterozygosity indices: H, SH and IR are calculated over all loci, while H', SH' and IR' are calculated without OHW loci and K31. With either heterozygosity index, the structure of the selected model was: A+H+R+AH+HR+AHR+A^2 ^plus the random effect of chick identity. Since the interaction A*H*R was significant, we also tested the significance of the A*H effect in each rank (A or B-chicks). P-values below 0.05 are in bold. *(Note: a more complete version, with the mean effect size for heterozygosity for each analysis, is given as additional file *1).

### Local effects and global effects of microsatellite loci

For mating patterns in 2003, we found that pairs were formed of genetically dissimilar individuals according to three of the 10 loci (*Phm *index for K32, K6 and K71). Hatching success was negatively correlated to genetic similarity of pairs in 2003 for two loci (*Phm *index for K32 and RBG20). Chick heterozygosity and survival until 25 days (in 2005) were positively correlated only for RBG29 (*H *index, p = 0.027; p > 0.1 for all other loci). For chick growth, we found that the parameters associated with the interactions Age*Heterozygosity and Age*Heterozygosity*Rank were significant in explaining chick growth in body weight and wing length when chick heterozygosity was estimated through three of the nine loci (*H *index for K16, K31, K32). Similarly, the parameters associated with the interactions Age*Heterozygosity and Age*Heterozygosity*Rank were significant in explaining chick growth in tarsus length when chick heterozygosity was calculated with two loci (*H *index for K31 and K32).

## Discussion

Our main objective was to examine predicted relationships between genetic variables and mating patterns in a genetically monogamous species. Selective pressures on mate choice may be stronger in such species, which do not mitigate the costs of homozygous offspring with extra-pair fertilizations. To our knowledge, there is only one other study of this kind of genetically monogamous species, the New Zealand robin (*Petroica australis*) and saddlebacks (*Philesturnus carunculatus*), in which pairings were random in relation to genetic similarity of mates [37]. Our findings in kittiwakes therefore appear to be the first evidence in a strictly monogamous species for the genetic similarity avoidance hypothesis.

Indeed, we found that kittiwake breeders were not paired randomly, but with mates that were less genetically similar than expected by chance. Observed pairs had a lower probability of producing homozygous offspring than expected, a finding that fits the predictions of the "genetic similarity avoidance" hypothesis. In contrast, the "genetic similarity preference" hypothesis predicted opposite results, and the "preference of heterozygous mates" hypothesis predicted assortative mating according to heterozygosity, a pattern we did not find.

Our findings show that genetically monogamous species may avoid pairing with genetically similar mates. Non-random mating with respect to genetic similarity has been reported in three shorebird species [14], ruffs (*Philomachus pugnax*, [11]), sand lizards (*Lacerta agilis*, [12]), mice [38] and humans [39]. In contrast, some other species behave consistently with the "good-genes as heterozygosity" hypothesis (e.g. house sparrows *Passer domesticus*, [7], and Seychelles warblers *Acrocephalus sechellensis*, [40]), or with the "genetic similarity preference" hypothesis (e.g. pied flycatcher *Ficedula hypoleuca*, [41], and great frigate birds *Fregata minor*, [10]), while other species display no significant relationships between mating patterns and genetic dissimilarity indices (e.g. great reed warbler *Acrocephalus arundinaceus*, [42]).

As expected, we also found deleterious effects of genetic similarity and homozygosity of offspring. This set of results (offspring hatchability, growth and survival) is statistically independent of results about mating patterns, but are biologically linked and consistent. Genetically similar pairs hatched fewer offspring than more dissimilar pairs (Figure 2), which is similar to studies that found significant effects of inbreeding on egg hatchability [29,30]. Likewise in these studies, hatching success was found to correlate negatively with pair genetic similarity. This pattern could be due to at least two non-exclusive effects. First, because both sexes incubate, cooperation between mates may be maximized when the genetic quality of the pair is high. Second, the overall genetic quality of heterozygous offspring may be higher and thus increase the success of early development. Given the relatively weak relationships between measures of reproductive success and genetic similarity, further evaluations of these hypotheses are warranted. Another apparent cost of homozygosity is that more homozygous offspring were less likely to reach 25 days of age than less homozygous ones. Again this pattern may be explained by different effects. For example, more heterozygous individuals may cope better with pathogens [8]. Furthermore, homozygous offspring are more likely to be produced by genetically similar pairs that may invest less in chick rearing, thus reinforcing the deleterious effects of homozygosity on chick growth and survival. We also found that chick growth slowed as homozygosity increased, which may also explain the observed differences in survival: slower growing chicks may be in worse condition than faster growing chicks and are thus less efficient at defending themselves against pathogens and environmental stress. Interestingly, this effect was only evident in second hatched chicks (Figure 4), which might be explained by the fact these chicks suffer from more severe selective pressures due to sibling competition and brood reduction (unpublished data, see also [43]).

The use of multi-locus estimates of heterozygosity and genetic similarity has been criticized by authors arguing that global heterozygosity-fitness correlations may be driven by certain loci that are physically linked to fitness-affecting loci [35,36,44]. However, such local effects are expected to be weak in this population, since we found no locus that correlated with all components of fitness. We are also aware that the number of microsatellites we used (7–10) is low given that the number of loci needed to achieve an accurate estimation of individual global heterozygosity may be much higher [45]. However, a small number of microsatellite loci should diminish our capacity to detect any relationship between heterozygosity and fitness components, making our analyses conservative. The fact that we found negative effects of genetic similarity and homozygosity on different components of fitness (mate choice, hatching success, offspring growth and survival) despite our relatively small number of loci suggests that genetic similarity is costly, and therefore selected against, in this population.

Little is known about mate choice in kittiwakes, and the pattern we describe here may result from different mechanisms. It could be due to a passive process with, for example, genetically similar pairs being more likely to fail in their breeding attempt, which in turn would make them more prone to divorcing than successful pairs. In our population, this seems unlikely since divorce after reproductive failure is not systematic [25]. Furthermore, the correlation between genetic similarity of mates and reproductive success may not be strong enough to make divorces significantly correlated to genetic similarity. Alternatively, kittiwakes may choose to pair with individuals from different areas within a structured population, but we think this is unlikely. Genetic structuring is low at the scale of the whole North Atlantic kittiwake population [46,47]). At a local scale, movements due to re-nesting of previously failed breeders into new areas are likely to diminish preexisting genetic structuring in this population. A third mechanism could be that prospective breeders may actively search for genetically dissimilar mates. The evolution of this strategy could be driven by the fitness costs of genetic similarity. Active mate choice would allow individuals to encounter a more adaptive mate faster than a passive process. However, experiments are needed to demonstrate that active mate choice has produced the observed pattern. Long-term monitoring and genotyping may also allow us to compare pairs that divorced with pairs that stayed together in relation to the genetic similarity of the first and second mate.

Active choice of genetically dissimilar mates has been demonstrated in mammals (e.g., humans, [48], mice [49] or fur seals, [50]) where genetic similarity is detecTable 2ecause of correlations between MHC alleles and body odors (humans, [51]; lemurs, [52]; mice [53]). Although the use of smell remains poorly known in birds, Antarctic prions (*Pachiptila desolata*) have been shown to recognize their mates through odors [54]. Genetically driven odors have not yet been shown in birds, but various aspects of social interactions in kittiwakes may allow them to recognize and choose their mates according to odor. Experiments are needed to determine the potential mechanisms for birds to estimate their relatedness to potential mates.

## Conclusion

Black-legged kittiwakes pair with individuals that are genetically more dissimilar than expected by chance, a pattern that is consistent with our observation of the existence of potential costs of producing homozygous progeny, such as decreases in hatching success and survival. In such genetically monogamous species, mate choice therefore seems to be under selection. Thus, the genetic criteria of mate and extra-pair mate choice in various genetically polygamous species may also be a general feature in genetically monogamous species. Indeed, breeders of strictly monogamous species may experience the highest selective pressure to choose genetically dissimilar mates.

## Methods

### Study species and population

We conducted our study on Middleton Island (Gulf of Alaska, 58°25' N, 146°19' W, May-July 2003–2006). This island supports a large declining population of black-legged kittiwakes (from 166,000 birds in 1981 to fewer than 25,000 in 1999; [55]). We studied kittiwakes nesting on the ledges of an abandoned U.S. Air Force radar tower that has been modified to enable close observations and easy capture. The study plot is characterized by vertical walls and uniform nest spacing, with breeders nesting on wooden ledges built specifically for cliff-nesting seabirds [55].

Nest sites were observed twice daily from mid-May to late-August to assess individual attendance and reproductive success. We arrived at the field site too late to assess the arrival dates of adults. During each visit, we recorded the color-band combination of attending adults, the stage of nest building, the presence of eggs and chicks and the behavior of adults in the form of incubation, nest building and copulation, variables that we used to confirm pair identity. Each chick was marked at hatching and banded at 25 days old. Adults received five bands (one coded U.S. metal and four color-bands). We could thus precisely determine laying and hatching dates of focal pairs, as well as the hatching rank of chicks (recorded as A for first hatched and B for second hatched; brood size rarely exceeds two chicks). Copulation behavior was also monitored in order to determine the sex of every paired adult; for unpaired adults, the sex was identified by morphological values such as bill length, tarsus length, bill width and head+bill length [56]. We sampled blood from breeding and non-breeding individuals in 2003–2004 in order to analyze mating patterns. Results for mating patterns in 2005 were not analyzed because of other experiments performed in 2004 that affected mating patterns and divorce rates in subsequent years. Therefore, in 2005, we used our dataset to analyze the fitness costs of homozygosity in chicks hatched from pairs that were not manipulated in 2004 or 2005. In order to analyze the fitness costs of homozygosity, chicks of such pairs were blood-sampled at hatching and were weighed and measured (for wing and tarsus length) every 5 days from hatching to 25 days old. We did not remain at the field station long enough to record fledging success, which occurs between 35 and 40 days old. However, mortality is very low between 25 days old and fledging in this species, and thus survival at 25 days old is a reliable estimation of survival at fledging (unpublished data). Blood was kept in a preservation buffer solution (Longmire buffer, [57]) that allows the storage of samples without refrigeration.

### Genetic analysis

#### DNA extraction

Whole genomic DNA was extracted from each blood sample using a "salting out" protocol described in [58]), modified by substituting Pronase E for Protease K and incubating at 37°C in the lysis phase, and substituting 0.7 volumes of 2-propanol in place of 2 volumes of ETOH in the DNA precipitation phase. Genomic DNA extractions were quantified using fluorometry and diluted to 50 ng/μL working solutions.

#### PCR and electrophoresis

Samples were genotyped at 10 microsatellite loci. Loci K6, K16, K31, K32, K67 and K71 were first described in the black-legged kittiwake [59], see Table 3); RBG20, RBG27, RBG29 and RBG39 were developed from the red-billed gull, *Larus novaehollandiae scopulinus *[60], see Table 3). Three additional loci (K56, [59], RBG18 and RBG13, [60]) were also tested but not used because the first two were found to be unreliable (see the quality controls below), and the third was monomorphic in our population.

**Table 3:** Summary of the ten microsatellite loci.

Locus	Repeated motif	Allele Sizes	No. of alleles	No. of ind.	H_exp_	H_obs_	p-value	Genebank Accession No.
K6	(AC)_4_T(TA)_12_	111–139	15	593	0.86	0.82	N.S.	AY083596

K16	(TG)_4_(TA)_8_(GA)_10_	151–187	13	591	0.86	0.72	< 0.0001	AY083597

K31	(TG)_13_	176–225	26	580	0.88	0.87	N.S.	AY083598

K32	(GA)_2_(GT)_12_	116–188	35	596	0.90	0.90	N.S.	AY083599

K67	(CA)_2_(TA)_9_	135–147	7	463	0.48	0.43	N.S.	AY083601

K71	(AC)_11_	143–159	7	593	0.65	0.69	N.S.	AY083602

RBG20	(GT)_13_	186–199	10	572	0.68	0.67	N.S.	AY091849

RBG27	(GT)_12_	207–223	9	593	0.73	0.71	N.S.	AY091851

RBG29	(GT)_13_	151–169	9	584	0.65	0.63	0.005	AY091853

RBG39	(AC)_11_	180–190	6	597	0.55	0.50	N.S.	AY091852

K6, K16, K31, K32, K67 and K71 were first described in the black-legged kittiwake [59]), whereas RBG20, RBG27, RBG29 and RBG39 were sequenced from the red-billed gull, *Larus novaehollandiae scopulinus *[60]). H_exp _and H_obs _are the expected and observed heterozygosities computed by GENEPOP, and we give also the p-value the Hardy-Weinberg equilibrium test (after Bonferroni correction for multiple tests).

The forward primer in each primer pair was synthesized with a modified 19- to 20-bp universal tail (M13F, M13R or SP6) added to the 5' end of the oligonucleotide [61]. We used a complementary fluorescently labeled (IRD700 or IRD800) primer, identical to the specific tail used to modify the forward primer, to detect alleles at each loci. We carried out amplifications in a final volume of 10 μL that contained 50 ng DNA extract, 0.2 mM dNTPs, 0.1 mg BSA, 1× PCR buffer (Perkin Elmer Cetus I; PE Biosystems, Forest City, California), 10.0 pmoles forward and reverse unlabeled primers, 1.0 pmole fluorescently labeled primer, and 0.2 units of Taq polymerase. PCR reactions began at 94°C for 90 seconds, and continued with 40 cycles each of 94 C for 15 s, 50°C for 15 s and 74°C for 30 s.

Amplification products were separated on a 48-well 25-cm 6% polyacrylamide gel on a LI-COR 4200 LR automated sequencer, using Base ImagIR™ (LI-COR, Inc., Lincoln, Nebraska). Allele sizes for specific samples at each locus were determined relative to the M13 phage single nucleotide ladder. These samples were later used as internal size standards to score new genotypes, using Gene ImagIR™ 4.05 software (Scanalytics, Inc., Fairfax, Virginia). For quality control purposes, we reprocessed a minimum of 10% of the samples for all markers, and only kept markers giving reliable scores. Two markers (K56, [59], and RBG18 [60]) that gave inconsistent scores at each PCR were thus discarded.

#### Genetic Diversity and Tests of Equilibrium

Mean number of alleles (A) and observed and expected heterozygosities (H_O _and H_E_) were calculated in GENEPOP Version 3.1 [62]. We also used this program to test linkage disequilibria and deviation from Hardy-Weinberg equilibrium (Markov chain parameters: 10,000 dememorization steps, 100 batches, and 5,000 iterations per batch). These different tests guided final marker selection.

A total of 645 adults were genotyped in the Middleton population from 2003 to 2006. The number of alleles per locus varied from 6 to 35. After correcting for multiple tests, two loci appeared to be out of Hardy-Weinberg equilibrium: K16 (p < 0.0001) and RBG29 (p = 0.005). Because of its lower heterozygosity, K67 was not genotyped in all samples. Thus, for each analysis, we present the results obtained using all loci, or excluding K16, K67 and RBG29, which are designated as the "OHW" loci, for "out of Hardy-Weinberg" equilibrium. Only K31 and K32 were genetically linked (p < 0.05 after correcting for multiple tests). Since our genetic similarity analyses are based on bootstrapping on the multi-locus genotypes (such that linkages between alleles are conserved for every bootstrap) and not on alleles, both loci were kept. We also conducted every allelic-based analysis with all ten loci and without "OHW" loci and K31 (7 remaining loci).

#### Genetic similarity indices

The genetic similarity between two given pair members (x and y) was estimated by the probability for this pair of producing homozygous offspring (*Phm*). For each locus (*l*), this probability is equal to:



following Lynch & Ritland's [63]) notation, where *s*_*ij *_is a Boolean factor equal to 1 if alleles *i *and *j *are similar (*i *and *j *standing for any other letter), and 0 otherwise. An index based on such probabilities was first proposed by Mathieu et al. [64], but has seldom been used. Belkhir et al. [65] showed that when the number of alleles per loci is low, such indices have a low variance in their estimations of relatedness, a property relevant to our study in which some loci were not highly variable (see Table 3).

To obtain a value for *Phm *across all loci, a weighted average of the *Phm*_*xy*_*(l) *is calculated using the formula proposed by Mathieu et al. [64]:



where *p*_*l *_is the probability of an individual being homozygous by chance at locus *l*. Therefore *Phm*_*xy *_will be close to 1 if the pair has a high probability of producing a homozygous offspring (pair members are closely genetically similar), and close to 0 otherwise.

#### Individual heterozygosity

We used three different indices to estimate individual global heterozygosity: the direct heterozygosity *H *(proportion of heterozygous loci in a given individual), the standardized heterozygosity *SH *and the internal relatedness *IR*, all described in [66]). All these indices were calculated using the same program computed in Delphi (Borland Delphi 5.0, ^©^1983, 1989 Inprise Corporation; Additional file 2 and 3).

#### Monte-Carlo analyses

Genetic indices were performed using a program computed (Additional file 2 and 3) in Delphi (Borland Delphi 5.0, ^©^1983, 1989 Inprise Corporation) that was previously tested for no significant differences with Queller & Goodnight estimations obtained from the IDENTIX software [65]. This program allowed us to calculate *Phm*_*xy *_and, given the configuration of our datasets, it makes our Monte-Carlo analyses much easier than previously published programs.

For each given sample of individuals, we re-mated adults randomly according to their sex 10,000 times (i.e. 10,000 bootstraps). These bootstraps gave a set of values that we used as the distribution under the assumption of random mating (in further analyses, we only give the mean of the genetic indices of all these bootstraps). The observed values of the genetic indices were then tested for significant departure from the expected distribution obtained by the bootstraps. When bootstrapping, we used only adults seen in the considered year, and in each bootstrap we randomly made the same number of pairs that had been genotyped in the population using all live adults, as they were all considered as potentially available for pairing. Allelic frequencies were calculated for this sub-sample of individuals. For Hypothesis 2, that mates are genetically less similar than expected by chance, statistical significance was assessed by the proportion of the bootstraps having a mean genetic index lower than the observed mean. We statistically tested Hypothesis 3 that mates share more alleles than expected by chance, by assessing the proportion of the bootstrap values with a mean genetic similarity index higher than the observed mean (i.e. 1 minus the p-value calculated under Hypothesis 2). By bootstrapping on observed individuals and not on randomly created individuals generated via allelic frequencies, we might also expect to correct for biases due to linkage disequilibria or genotyping errors. Individuals with incomplete genotypes had thus the same probability of being included in observed pairs as in bootstrapped pairs. Thus, biases will be similar for observed and bootstrapped pairs.

### General statistics

General statistical analyses were made using SAS^® ^package (^©^SAS Institute Inc.1999, Cary, NC, USA). Correlations between the two indices of genetic similarity, or between male and female heterozygosity, were analyzed through standard linear models. To analyze the links between reproductive success (number of eggs laid and the number and proportion of eggs hatched) and genetic variables, we used a generalized linear model (allowing fitting standard linear models to discrete variables) since the dependent variables never showed more than three levels. For each of these three levels, the genetic variables and the residuals from the model were normally distributed. Similarly, we analyzed the correlations between chick survival and heterozygosity using generalized linear models, since both chick survival (alive or dead) and hatching rank (A or B) are binary effects. For each rank and each survival value, the residuals from the model procedure were normally distributed.

For the analyses of growth variables (body weight, tarsus or wing length), we have repeated measures of individuals, having measured each chick every 5 days. We therefore conducted mixed linear models with chick identity as a random parameter in order to account for non-independence. We made mixed linear models with tarsus length, wing length or body weight as dependent variables, chick identity as a random parameter, and chick age (A), age*age (A^2^), heterozygosity (H) and hatching rank (R) as explaining variables. We added A^2 ^because growth of morphological traits is non-linear. For each variable and each heterozygosity index, we ran several models and kept only the one with the lowest AIC value. Given that the three growth variables are correlated, and given that the different indices of heterozygosity are also correlated, the structure of the model with the lowest AIC was always the same, in the form of Growth variable = A + H + R + A*H + A*R + H*R + A*H*R + A^2 ^+ Chick identity as a random parameter. We will only give the results obtained with the latter model, since the difference in AIC between this model and other models was always higher than 2. The significance of the interaction A*H was then tested in each rank separately.

## Authors' contributions

HM carried out the genetic analyses, monitored the birds on Middleton Island and drafted the manuscript with RHW. ED and RHW conceived the study and participated in its design and coordination. JFW and FH helped in monitoring the birds and in the conception of the study in the field. SLT and AMR participated in the microsatellite genotyping and scoring. SAH is responsible of the long-term monitoring on Middleton Island and gave access to the study area. All authors read and approved the final manuscript.

## Supplementary Material

Additional file 1 **Table **2**with effect size of heterozygosity**. Extended version of the Table 2, including mean effect size of heterozygosity for all analyses. Click here for file

Additional file 2 **Code of the program Nausicaa**. This file contains the complete code of the program used for calculation of genetic indices and Monte-Carlo analyses. Click here for file

Additional file 3 **GNU-GPL Licence**. GNU-GPL Licence for the program Nausicaa contained in additional file 2. Click here for file
