# Supplementary material for: Evidence that pairing with genetically similar mates is maladaptive in a monogamous bird
Source: BMC Evol Biol. 2009 Jun 30;9:147. doi: 10.1186/1471-2148-9-147 (PMC2709659; doi:10.1186/1471-2148-9-147)
Supplement: Additional file 1 — Table 2with effect size of heterozygosity. Extended version of the Table 2, including mean effect size of heterozygosity for all analyses. [file 1471-2148-9-147-S1.doc]

**Table 3: Relationships between chick age, hatching rank, heterozygosity and growth in body weight, wing and tarsus length.**

| Explained variable | Hz index used | A*H*R | AIC | A*H in A-chicks | A*H in B-chicks | Effect size of Heterozygosity | | | | | | | | | | | |
| --- | --- | --- | --- | --- | --- | --- | --- | --- | --- | --- | --- | --- | --- | --- | --- | --- | --- |
|  |  |  |  |  |  | A-chicks | | | | | | B-chicks | | | | | |
|  |  |  |  |  |  | 0 days  (n=53) | 5 days  (n=45) | 10 days  (n=40) | 15 days  (n=37) | 20 days  (n=37) | 25 days  (n=37) | 0 days  (n=28) | 5 days  (n=20) | 10 days  (n=11) | 15 days  (n=10) | 20 days  (n=7) | 25 days  (n=7) |
| *Body weight* | *H* | **<0.0001** | 3267.5 | 0.68 | **<0.0001** | 0.0071 | 0.11 | 0.14 | 0.077 | 0.067 | -0.024 | -0,10 | 0.023 | 0.45 | 0.72 | 0.42 | 0.63 |
|  | *SH* | **<0.0001** | 3271.7 | 0.44 | **<0.0001** | 0.010 | 0.13 | 0.16 | 0.13 | 0.12 | -0.0090 | -0.11 | 0.051 | 0.39 | 0.68 | 0.35 | 0.59 |
|  | *IR* | **<0.0001** | 3273.3 | 0.86 | **<0.0001** | -0.0034 | -0.083 | -0.12 | -0.034 | -0.025 | 0.039 | 0.067 | -0.037 | -0.42 | -064 | -0.39 | -0.64 |
|  | *H’* | **0.020** | 3284.8 | 0.12 | **0.0020** | 0.062 | 0.18 | 0.26 | 0.22 | 0.24 | 0.058 | -0.085 | 0.084 | 0.23 | 0.54 | 0.064 | 0.30 |
|  | *SH’* | **0.046** | 3285.9 | 0.094 | **0.0038** | 0.060 | 0.18 | 0.27 | 0.25 | 0.29 | 0.049 | -0.055 | 0.12 | 0.18 | 0.49 | 0.070 | 0.33 |
|  | *IR’* | 0.064 | 3289.2 | 0.12 | **0.0055** | -0.050 | -0.14 | -0.23 | -0.19 | -0.20 | -0.066 | 0.094 | -0.093 | -0.18 | -0.47 | -0.029 | -027 |
| *Wing length* | *H* | **<0.0001** | 2612.3 | 0.58 | **<0.0001** | -0.10 | -0.018 | 0.075 | 0.10 | -0.078 | -0.093 | -0.16 | -0.26 | 0.18 | 0.68 | 0.50 | 0.56 |
|  | *SH* | **0.0001** | 2615.0 | 0.91 | **<0.0001** | -0.12 | 0.00055 | 0.10 | 0.15 | -0.052 | -0.062 | -0.15 | -0.26 | 0.14 | 0.64 | 0.48 | 0.52 |
|  | *IR* | **<0.0001** | 2615.4 | 0.39 | **<0.0001** | 0.16 | 0.050 | -0.062 | -0.077 | 0.11 | 0.12 | 0.20 | 0.20 | -0.17 | -0.63 | -0.50 | -0.55 |
|  | *H’* | **0.013** | 2621.7 | 0.31 | **0.0007** | -0.11 | 0.046 | 0.29 | 0.27 | 0.046 | 0.020 | -0.049 | -0.20 | 0.036 | 0.55 | 0.38 | 0.34 |
|  | *SH’* | **0.037** | 2621.6 | 0.15 | **0.0013** | -0.14 | 0.048 | 0.31 | 0.30 | 0.075 | 0.050 | -0.032 | -0.18 | 0.0094 | 0.49 | 0.38 | 0.35 |
|  | *IR’* | **0.038** | 2626.8 | 0.41 | **0.0033** | 0.14 | 0.028 | -0.27 | -0.23 | -0.0073 | -0.0068 | 0.13 | 0.16 | 0.026 | 0.50 | -0.31 | -0.28 |
| *Tarsus length* | *H* | **0.0017** | 2758.8 | 0.71 | **0.0025** | -0.14 | 0.049 | 0.020 | -0.016 | -0.16 | -0.16 | 0.065 | -0.61 | 0.23 | 0.77 | 0.41 | 0.30 |
|  | *IR* | **0.0013** | 2760.4 | 0.95 | **0.011** | -0.17 | 0.046 | 0.054 | 0.038 | -0.10 | -0.14 | 0.095 | -0.58 | 0.14 | 0.71 | 0.39 | 0.27 |
|  | *SH* | **0.012** | 2762.2 | 0.28 | **0.0045** | 0.11 | -0.018 | 0.00089 | 0.085 | 0.26 | 0.24 | -0.080 | 0.57 | -0.19 | -0.73 | -0.33 | -0.34 |
|  | *H’* | **0.027** | 2765.2 | 0.75 | **0.04** | -0.069 | 0.11 | 0.071 | 0.041 | -0.095 | -0.13 | -0.11 | -0.42 | -0.027 | 0.64 | 0.31 | -0.051 |
|  | *IR’* | **0.014** | 2767.2 | 0.20 | 0.054 | -0.097 | 0.098 | 0.12 | 0.083 | -0.041 | -0.11 | -0.062 | -0.36 | -0.085 | 0.55 | 0.31 | -0.0059 |
|  | *SH’* | 0.089 | 2766.3 | 0.97 | 0.089 | 0.013 | -0.064 | -0.037 | 0.019 | 0.23 | 0.22 | 0.092 | 0.41 | 0.074 | -0.61 | -0.21 | 0.081 |

We used AIC for model selection. Parameters: A (Age), H (Heterozygosity, estimated by the index in the column “Hz index used”) and R (hatching Rank, a binary effect). For heterozygosity indices: H, SH and IR were calculated over all loci, while H’, SH’ and IR’ were calculated without OHW loci and K31. With either heterozygosity index, the structure of the selected model was: A+H+R+AH+HR+AHR+A² plus the random effect of chick identity. Since the triple interaction A*H*R was significant, we also tested the significance of the A*H effect in each rank (A or B-chicks). P-values below 0.05 are in bold. The 12 last columns display the mean effect size for heterozygosity (H), estimated as the R coefficient of Pearson, for each rank and each age (every chick was weighed and measured every 5 days from hatching to 25 days old). The number in brackets in the third line (n) record the number of chicks included in each analysis (mortality is high during the first days post-hatching).
